# Supplementary material for: Substantial terrestrial carbon emissions from global expansion of impervious surface area
Source: Nat Commun. 2024 Jul 31;15:6456. doi: 10.1038/s41467-024-50840-w (PMC11291968; doi:10.1038/s41467-024-50840-w)
Supplement: Supplementary file 1 — Supplementary Information [file 41467_2024_50840_MOESM1_ESM.pdf]

Supplementary Materials for

Substantial terrestrial carbon emissions from global expansion of impervious surface area

Linghua Qiu[1,2,*], Junhao He[3,*], Chao Yue[4,1,3], Philippe Ciais[5], Chunmiao Zheng[6,2]

[1] Shenzhen Research Institute, Northwest A&F University, Shenzhen 518000, China
[2] School of Environmental Science and Engineering, Southern University of Science and Technology, Shenzhen 518055, China
[3] State Key Laboratory of Soil Erosion and Dryland Farming on the Loess Plateau, Institute of Soil and Water Conservation, Northwest A&F University, Yangling, Shaanxi 712100, China
[4] College of Natural Resources and Environment, Northwest A & F University, Yangling, Shaanxi, China
[5] Laboratoire des Sciences du Climat et de l'Environnement, LSCE/IPSL, CEA-CNRS-UVSQ, Université Paris-Saclay, 91191 Gif-sur-Yvette, France
[6] Eastern Institute for Advanced Study, Eastern Institute of Technology, Ningbo 315200, China
[*] These authors contributed equally to this work.
Correspondence: Chao Yue (chaoyuejoy@gmail.com)

Email addresses: qiulh2022@gmail.com (Linghua Qiu), junhaohe@nwafu.edu.cn (Junhao He), chaoyuejoy@gmail.com (Chao Yue), philippe.ciais@cea.fr (Philippe Ciais), zhengcm@sustech.edu.cn (Chunmiao Zheng)

Supplementary Information

Supplementary Information 1 | Feasibility of assuming 100% ISA coverage for the mapped ISA pixels

In this study, we assume that all impervious surface or urban area products have 100% of ISA coverage for the mapped ISA or urban pixels, irrespective of the naming of the land-cover type concerned (i.e., 'impervious surface' or 'urban'). The plausibility of this assumption is based on three considerations: (1) Landscape homogeneity is the key assumption underlying the classification of land cover and its subsequent spatial mapping and this assumption is best guaranteed through high-resolution satellite mapping, which is the case for most ISA products (for GAIA, GAUD and GISA2.0, the spatial resolution is 30 m) used in this study. Following this assumption, it is a common practice for previous studies to treat the classified ISA pixels as being 100% ISA coverage. In this study, this assumption is further strengthened by the fact that all ISA products used have a strong tendency to ensure that the classified ISA (or urban) pixels are highly dominated by ISA through their mapping methodologies (detailed below). (2) Treating mapped ISA pixels as being 100% ISA coverage will not likely lead to an overestimation of global ISA. Although it is highly possible that the mapped ISA pixels can contain minor sub-pixel areas that are non-ISA, but these areas can be compensated by sub-pixel ISA areas present in non-ISA pixels. This claim could be supported by the fact that the ISA areas reported by the four ISA products for several cities (as case studies) are comparable to those estimated by using images of very high spatial resolutions that can be considered as ground truth. (3) The global ISA expansion estimated here is more likely underestimated, rather than overestimated, considering that ISA covered by global minor roads is very likely omitted in the ISA products used in this study.

1.1 Mapped ISA or urban pixels are highly dominated by ISA supporting the assumption of being 100% coverage of ISA

Landscape homogeneity is the key assumption underlying the classification of land cover and its subsequent spatial mapping. In case of land cover mapping through using satellite images, pixels of a given spatial resolution with similar spectral signatures are typically identified as having the same land cover. The validity of the landscape homogeneity assumption is best guaranteed through using high-resolution satellite images because they can distinguish land cover distributions in spatially heterogeneous mosaic lands. Of the four ISA or urban products used in this study, three (GAIA, GAUD, GISA2.0) have a 30-m resolution and support this assumption well. The ESA CCI product has a comparable ISA expansion with the three 30-m products despite having a medium resolution of 300 m (detailed below). Following the landscape homogeneity assumption, previous studies on land use and land cover change almost universally assumed that a satellite pixel classified as a given land cover type has a 100% coverage of that land cover type [1–3]. Likewise, previous studies focusing on global and regional ISA expansion also assumed that the classified urban or ISA pixels have a 100% coverage. For example, ref. [4] estimated the terrestrial NEP loss due to ISA expansion by multiplying ISA area (obtained from GISA) with the NEP loss per area, clearly assuming that a pixel transitioning to ISA lost 100% of its original NEP. Ref. [5] overlayed carbon density map with urban expansion map to calculate the loss in vegetation biomass, also

assuming a pixel transitioning to urban land lost 100% of its original biomass. Both studies hence assumed that the classified ISA or urban pixels are 100% covered by ISA.

Below we further elaborate on the fact that the mapping methodologies of the four ISA products used in this study ensure that their mapped ISA or urban pixels are highly dominated by ISA, supporting our assumption that the classified ISA or urban pixels are 100% covered by ISA. The ISA mapping methodologies include mapping data sources, training sample collection and classification methods, all of which can influence the performance of the ISA products.

(1) The GAIA product is generated using the full archives of 30-m Landsat Thematic Mapper (TM), Enhanced Thematic Mapper Plus (ETM+) and Landsat 8 Operational Land Imager (OLI) time series data, which facilitate high-resolution and long-term global ISA mapping. These Landsat imageries are also fundamental data sources for GAUD and GISA2.0. Training samples for GAIA are collected from earlier Landsat images (circa 1985) using high-resolution Google Earth images as guidance, under the assumption that the developed ISA is irreversible and will expand spatially over time, hence leading to growing training sample size for subsequent years[6]. Then GAIA employed an "exclusion-inclusion" mapping approach. It first identifies and excludes pixels that are definitively not covered by ISA, and then applies a quantitative similarity assessment over the remaining pixels to determine ISA presence, assisted by the training samples and predictive remote sensing variables including Normalized Difference Vegetation Index (NDVI), Visible Infrared Imaging Radiometer Suite (VIIRS) nighttime light (NTL), shortwave infrared (SWIR), modified normalized difference water index (MNDWI), and Sentinel-1 Synthetic Aperture Radar (SAR). Their mapping approach assumes that the classified ISA pixels are locally homogeneous and contain an ISA fraction greater than 50%. To further ensure that the mapped pixels are dominated by ISA, GAIA excludes highly reflective areas and vegetated areas, such as airport, roads, parks or built-up areas under trees, given their different spectral signatures from built-up environments.

(2) GAUD is produced by fusing the GAIA, the Global Human Settlement Layer (GHSL)[7], the Global urban land (GUL)[8], and the Global Urban Footprint (GUF)[9]. The later three products delineate global bult-up areas, defined as 'any roofed structure erected above ground for any use', which are clearly impervious surfaces. GAUD identifies the pixels that are consistently mapped as ISA by all input ISA products as high-confidence ISA areas, while the areas with inconsistent mapping by the input ISA products are identified as potential ISA areas, which are then reclassified as urban or non-urban using training samples collected from the high-confidence areas. Moreover, the GAUD's author team studied 'urban green recovery' during their study period of 1985–2015, a phenomenon characterized by built-up areas reverting to more vegetation-dominated states but remaining classified as the 'urban' land cover type. However, according to their results, the area undergoing 'urban green recovery' is two orders of magnitude smaller than the area of built-up area expansion during the same time (Fig. 3 in ref. [10]), suggesting that only a tiny fraction of their 'urban' areas had a noticeable vegetation signal. Therefore, we consider GAUD to be dominated by ISA rather than by urban green areas.

(3) GISA2.0 employs four high-resolution global ISA products: GISA1.0[11], GAIA, GHSL, and GAUD. The GISA2.0 author team utilizes an approach that first divide the global terrestrial surface into grids, and for each grid, the ISA density is calculated as the ratio of consistent ISA of

the four products to the grid area. Then, the top 10% grids having the highest ISA density are selected for ISA mapping. Subsequently, two categories, namely A-Grids and M-Grids, are determined based on the degree of consistency among four ISA products, with the A-Grids exhibiting better accuracy and reliability due to the greater agreement among four ISA products compared to the M-Grids. Different training sample collection approaches are used in these two categories. More specifically, for the A-Grids, an automatic mapping approach is applied to the inconsistent ISA pixel regions among four ISA products, using the ISA and non-ISA training samples collected from the consistent ISA and consistent non-ISA regions among four ISA products. For the M-Grids, two types of training samples are utilized: the first type contains the training samples same as those used in A-Grids; and the second type consists manually-interpreted samples located at regions with ISA omissions and inconsistencies among four products, which can help to correct the classification errors and reduce the uncertainties. Eventually, the random forest classifier is adopted for these two regions for ISA classification.

(4) The mapping of "urban area" land cover type in the ESA CCI product is based on two external datasets: GHSL and GUF, according to the product user guide (https://climate.esa.int/media/documents/CCI_Land_Cover_PUG_v2.0.pdf). Both datasets clearly focus on mapping ISA. The fact that they are also applied in GAUD and GISA2.0 indicates a strong conceptual coherence among GAUD, GISA2.0 and the ESA CCI product, justifying our choice to use ESA CCI along with the other 30-m resolution ISA products.

All four ISA products have been evaluated in terms of their overall accuracy (OA). The OA values reported for GAIA, GAUD, and GISA2.0 were 94%, 79%, and 97.89%, respectively. However, due to the different methods in validation sample collection, their reported OAs are not fully comparable. An independent study validated existing ISA products using multiple sources of manually interpreted, multi-temporal and high-resolution ISA samples[12]. The OA values for GAIA, GAUD, and GISA2.0 were 87.61%, 91.18%, and 92.97%, respectively, indicating that these products can well display global ISA extents. A regional assessment study reported that the ESA CCI product achieved an OA value of 85.1% in mapping China's rural settlement (i.e., impervious surfaces)[13]. Therefore, we conclude that these four ISA products are suitable for investigating the dynamics of global ISA expansion.

Last, all four ISA products have good consistency with each other in terms of the area of ISA expansion, with GAIA, GAUD, GISA2.0, and ESA CCI showing 0.34 Mkm^2, 0.27 Mkm^2, 0.29 Mkm^2, and 0.37 Mkm^2, respectively, for the overlapping period of 1996–2015 (Supplementary Fig. 2). Such a high consistency is very likely a result of their high consistency in ISA mapping.

1.2 Mutual compensation between sub-pixel ISA and non-ISA areas

Although each of the four products focuses on mapping the distribution of either ISA or 'urban area' which is dominated by ISA, we acknowledge that, given the high spatial heterogeneity of urban environments, the ISA pixels identified by these products might still contain sub-pixel urban green areas with a dimension much smaller than the spatial resolution of the products (30 m or 300 m). But on the other hand, the reverse phenomenon also happens, i.e., small ISA patches might also have been omitted in the identified non-ISA pixels in these products. We expect that

these two phenomena will partly offset each other and it is unlikely that assuming the mapped ISA or urban pixels as being 100% ISA coverage will overestimate global impervious surface area.

This supposition can be tested by comparing the ISA areas reported by these products with those based on remote sensing with a very high spatial resolution which could be considered as ground truth. For example, ISA in Mannheim, Germany, being estimated using very-high-resolution (0.1 m) images, accounted for 42.5% of the total area of urban environment[14], whereas the area percentage covered by ISA was 50.8%, 46.9%, 52.6%, and 53.3% according to GISA, GAIA, GAUD, and ESA CCI, respectively for the same region (with an average of 50.9%). Using 10-m resolution remote sensing, the ground coverage of ISA in two cities of Foshan and Wuhan, China, was reported as 53.3% and 51.2%, respectively[15], whereas according to the four ISA products, the ISA coverage was 54.3% (51.3%–56.4%) for Foshan and 43.8% (42.6%–45.9%) for Wuhan, respectively. Although we do not intend to provide an inclusive and complete validation for the ISA products used, these case studies show that the ISA areas mapped by the four products are comparable with the ground truth, with the error in estimated ISA ground coverage by averaging all the products not exceeding 10%.

1.3 Possible neglection of ISA covered by global minor roads

Roads are one of the most important components of ISA. Previous studies have reported that major roads could be reliably detected using remote sensing images with a 30-m resolution, but minor roads can rarely be detectable[16]. Minor roads are often located in the low-density ISA regions mingling with bare land or vegetation, leading to classification errors[17,12]. Hence, the impervious surface area of minor roads was very likely not included in the ISA products used in our study.

To get an idea of how much impervious surface area could have been omitted, we performed a rough estimation of the global ISA of minor roads using an up-to-date global road map recently released by the Global Roads Inventory Project (GRIP)[18]. GRIP provides global road lengths according to five identified road types across the globe: highways, primary roads, secondary roads, tertiary roads, and local roads. Globally, the total lengths are 0.7, 2.5, 4.9, 5.0, and 8.5 million km for the five road types, respectively. According to the road features available from OpenStreetMap (OSM, ref. [19]), highways and primary roads are mostly major roads which can probably be successfully mapped at 30-m resolution. Therefore, the remaining minor roads, consisting of secondary roads, tertiary roads and local roads, are considered as being omitted by the ISA products used in our study. According to the information available on the number of lanes and the lane width for each road type from OSM (https://planet.openstreetmap.org/, accessed 20 October 2022), the road widths are set as 9 m for secondary roads, 6 m for tertiary roads and 3 m for local roads. The total area of minor roads globally, calculated by multiplying the road lengths from GRIP by the assumed road widths, was thus estimated as 99410 km^2 in total, consisting of 44045 km^2 of secondary roads, 29717 km^2 of tertiary roads and 25649 km^2 of local roads.

The estimated area of global minor roads accounts for 18.4% of the total ISA expansion during 1986–2018 and 12.6% of the existing ISA in 2018 according to our study. The value of 18.4% represents an upper boundary for omitted ISA expansion contributed by minor roads for two reasons. First, the estimated minor road areas include all existing minor roads globally rather

than road expansions during 1986–2018. Second, the assumed road widths might best describe the situations in a few developed countries and are probably overestimates for many less developed countries. Nonetheless, if we do not account for such an overestimation in road widths and further assume that minor road networks have developed in proportion to the ISA expansion, then 12.6% is the percentage of ISA expansion due to minor roads that were omitted by the four products used in this study.

Supplementary Information 2 | The uncertainties in emissions induced by spatial interpolation of carbon densities

In this study, static AGB, BGB, and SOC density maps rather than dynamic maps due to data availability were used to estimate terrestrial carbon emissions induced by ISA expansion. However, the interpolated carbon densities rather than the original values in the intensive ISA pixels were used to address the concern of the alteration of carbon densities induced by the intensive ISA expansion caused by human activities. Essentially, landscape homogeneity assumption is the basis for interpolation of carbon densities. And we also employed this assumption to estimate the uncertainty induced by the carbon density interpolation. Below these details were described.

The static carbon density maps of biomass and SOC used in this study are mainly validated for homogeneous landscapes. For example, the static biomass density maps circa 2010 of ref. [20] reports biomass densities for different land cover types using the land cover map distribution from the ESA CCI product of 2010. In this study, carbon densities for pixels transitioning to ISA are primarily obtained from carbon density maps through identifying their land cover type information. Given that landscape homogeneity is the key assumption underlying the classification of land cover and its subsequent spatial mapping, we argue that obtaining carbon densities by combining carbon density maps and land cover maps is consistent with their underlying landscape homogeneity assumptions.

In developing the methods of this study, our experiences revealed that the fraction of ISA derived from high-resolution (30 m) ISA products within a 300-m ESA CCI pixel is an important factor to break the land homogeneity assumption described above. Unlike vegetated areas, conceptually ISA (e.g., ground covered by tar, concrete, asphalt or mixtures) has almost zero biomass. Therefore, the fraction of ISA within a 300-m pixel can highly influence its biomass density even if the whole pixel could still be identified as a vegetated pixel according to the ESA CCI product. Hence, to strengthen the validity of the homogeneity assumption, we deemed all carbo density observations for 300-m pixels with an existing ISA fraction exceeding 5% in 2018 (defined as ISA-contaminated pixels) as invalid. Carbon densities, including both biomass and SOC, for ISA-contaminated pixels, are interpolated using valid carbon densities from the 20 nearest ISA-free pixels, defined as pixels with an ISA fraction <5% or without any ISA coverage. The spatial interpolation was based on the assumption that, before any land conversion to ISA, neighbouring pixels of the same land cover type share similar carbon densities.

Carbon density interpolation will introduce uncertainty to the estimated carbon emissions caused by ISA expansion. As the (true) values of carbon densities for ISA-contaminated pixels under the land homogeneity assumption (i.e. before having any ISA) are unknown, the exact error caused by interpolation cannot be quantified. However, this error could be approximated by interpolating carbon densities for ISA-free pixels whose observed carbon densities (which could be considered as true values) are known and, therefore, no interpolation is needed.

Specifically, the ISA-free pixels around ISA-contaminated pixels, defined as buffer ISA-free pixels, were identified and their original carbon densities were considered as true values, whereas their interpolated carbon densities using carbon densities of the 20 nearest ISA-free pixels with the same land cover type with an inverse distance algorithm were considered as predicted values. Then the relative error (RE) between the interpolated and observed (true) carbon densities as:

$$RE = \frac{Interpolated - Oberved}{(Interpolated + Observed)/2} \times 100\% \qquad (S1)$$

This metric was selected for its symmetry so that the distribution of RE values will not be skewed. The median and standard deviation of RE values were then quantified for all the valid buffer ISA-free pixels at the global scale or for a given land cover type.

For biomass density interpolation, the average biomass density of three biomass density maps (i.e., refs. [20–22]) for forests and shrubland, and the static biomass density map from ref. [20] for cropland, grassland and wetland were combined to form a global biomass density map. This combined static AGB map was utilized to estimate the interpolation error for biomass density. The spatial distribution of RE values on a 5-km scale is shown in Supplementary Fig. 15. Spatially, the RE values were rather heterogeneous. In terms of the distribution of RE, 73% of RE values were found within the range of -10% to 10%, and 94% were within -30% to 30% (Supplementary Fig. 16b). At the global scale, the RE values, obtained by integrating all land cover types, is $+1.3^{+7.1}_{-2.0}\%$ (median, interquartile range of RE), suggesting a likely very small overestimation of ISA-driven biomass carbon emissions caused by biomass carbon density interpolation. The RE for different land cover types range from $+0.1^{+6.8}_{-5.1}\%$ for cropland to $+3.3^{+11.3}_{-1.1}\%$ for forest (Supplementary Fig. 16a).

RE caused by SOC density interpolation shows a relatively homogeneous spatial distribution (Supplementary Fig. 17). In terms of the statistical distribution of RE, 98.7% of RE values are found within the range of -5% to 5%, and 99.8% are found within the range of -10% to 10% (Supplementary Fig. 18b). The global RE for SOC interpolation is $+0.1^{+0.6}_{-0.4}\%$. RE is also very small across the different vegetation types (Supplementary Fig. 18a).

Although RE provides an approximate for the error in the interpolated carbon densities, the true value of error could not be known exactly. Hence, we avoid correcting the estimated ISA-driven emissions in 5-km grids by using the information of RE. Rather, the statistical distribution of RE was used to calculate the uncertainties of the derived ISA-driven biomass, SOC, and total carbon emissions. Since the ISA-driven carbon emissions are the products of carbon density maps and annual ΔISA maps, given that ΔISA is independent of the error in carbon density interpolation, RE values for carbon densities can then be directly applied on the estimated emissions to derive the interquartile range of emissions. Hence, we can consider that the estimated biomass carbon emissions have a RE of $+1.3^{+7.1}_{-2.0}\%$, and the estimated SOC emissions have a RE of $+0.1^{+0.6}_{-0.4}\%$. Re-arranging the Equation (S1) and applying the two quartile values of RE, we can derive the uncertainty of carbon emissions as $31.0^{31.6}_{28.9}$ Tg C yr^{-1} (our estimated carbon loss and interquartile range of estimated carbon loss) for biomass carbon loss, $43.9^{44.1}_{43.6}$ Tg C yr^{-1} for SOC loss of the upper boundary, and $14.8^{14.9}_{14.7}$ Tg C yr^{-1} for SOC loss of the lower boundary, respectively. Because the distribution of RE is represented by its interquartile range, these intervals for biomass and SOC losses suggest that there is a 50% probability that the true value of emissions will fall within these intervals. Furthermore, the uncertainty for global ISA-driven carbon emission can be calculated by simply adding the intervals of biomass with those of upper and lower estimates of SOC emissions, respectively. Therefore, the global total ISA-driven carbon emissions are $74.9^{75.7}_{72.5}$ Tg C yr^{-1} and $45.8^{46.5}_{43.6}$ Tg C yr^{-1} for the upper and the lower boundary estimations, respectively.

Supplementary Information 3 | The LMDI decomposition

The Kaya decomposition method is able to decompose the relative trend of a target variable into the aggregation effect of the relative trends of several underlying driving variables but with a residual. Alternatively, the logarithmic mean Divisia index (LMDI) approach, which can reach complete decomposition without leaving any residual[23], has also been widely applied in emission decomposition studies at global and national scales[24–26]. However, the disadvantage of LMDI is that only the change between two different years, rather than the trend over a given period, could be decomposed. More specifically, using the same underlying driving factors as in the main text Equation (3), the change of ISA-driven carbon emission between the final (E_t, E at 2018) and initial (E_0, E at 1993) years can be decomposed into the following additive form,

$$\begin{aligned} \Delta E = E_t - E_0 &= \Delta P + \Delta\left(\frac{Pu}{P}\right) + \Delta\left(\frac{ISA}{Pu}\right) + \Delta\left(\frac{\Delta ISA}{ISA}\right) + \Delta\left(\frac{E}{\Delta ISA}\right) \\ &= \Delta P + \Delta u + \Delta r + \Delta s + \Delta e \end{aligned} \quad \text{(S2)}$$

The decomposed variables in Equation (S2) can be obtained by the following equations,

$$\Delta P = L \cdot ln\,(\frac{P_t}{P_0}) \quad \text{(S3)}$$

$$\Delta u = L \cdot ln\,(\frac{u_t}{u_0}) \quad \text{(S4)}$$

$$\Delta r = L \cdot ln\,(\frac{r_t}{r_0}) \quad \text{(S5)}$$

$$\Delta s = L \cdot ln\,(\frac{s_t}{s_0}) \quad \text{(S6)}$$

$$\Delta e = L \cdot ln\,(\frac{e_t}{e_0}) \quad \text{(S7)}$$

where L is the weighted logarithmic mean of E_t and E_0 given by,

$$L = \frac{E_t - E_0}{ln\,(E_t)\ - ln\,(E_0)} \quad \text{(S8)}$$

As is mentioned above, the LMDI method reveals the decomposing changes of ISA-driven carbon emission into five factors between two individual years, whereas the variations within the time period between the initial and end years are omitted. For example, if the emission (E) or any of the contributing factors (*Purse*) shows no change between year 0 and year t but their time series over this period exhibit increasing or decreasing trends, LMDI will not capture any temporal trends. If the concerned variables show monotonous changes over time with a well-fitted linear trend, then the Kaya and LMDI methods will show highly consistent results.

Here, in order to use the LMDI method to independently verify the patterns of contributing factors to drive the temporal trend in ISA-driven emissions revealed by Kaya analysis, adaptions are made for the LMDI method to make it decompose for the comparable period for which the Kaya method is applied. More specifically, we calculated the linear predicted values for 1993 and 2018 for each variable in Equation (S2) using a linear regression model with time. The predicted values are denoted as: $\hat{E}_{1993}$, $\hat{E}_{2018}$, $\hat{P}_{1993}$, $\hat{P}_{2018}$, $\widehat{Pu}_{1993}$, $\widehat{Pu}_{2018}$, $\widehat{ISA}_{1993}$, $\widehat{ISA}_{2018}$, $\widehat{\Delta ISA}_{1993}$, and $\widehat{\Delta ISA}_{2018}$, which are then substituted into Equation (S2) to investigate the absolute contributions of different factors to the temporal linear trend of ISA-driven carbon emissions (i.e.,

$\Delta E = \hat{E}_{2018} - \hat{E}_{1993}$). Then, to make the results comparable with the relative trends as reported in the Kaya method, the absolute contributions are divided by the mean value of carbon emissions ($\bar{E}$) over 1993 to 2018 and then by the total number of years (i.e., 26 years) to obtain the annual relative contributions of different factors. The averaged results of LMDI approach were obtained from the upper- and lower-boundary estimations for global, the non-Annex I countries (NAI), and the Annex I countries (AI), respectively. Compared with the results from Kaya method, those from the LMDI approach exhibit identical patterns and magnitudes of the underlying driving factors (Supplementary Fig. 14). This implies that the residuals in the Kaya method are trivial to the interpretation of the decomposition results.

Supplementary tables and figures

Supplementary Table 1 Treatment of urban land-cover type in selected DGVMs. The information for each individual model was obtained by sending questionnaires to the contact scientist for each model.

Model	Description
JSBACH v3.2	Urban land is not explicitly represented but is aggregated with the pasture plant functional type.
LPX-Bern	A "fake" urban plant functional type (PFT) within an urban land-use class is used. The urban PFT parameter values are chosen such that no vegetation can be established.
OCN	Urban land cover type is treated as bare soil because impervious surface is treated as bare soil.
LPJ-wsl	Urban land is merged with cropland and then treated as cropland.
SDGVM	There is an urban land-cover type but it is not used for GCP global carbon budget simulations. Urban land cover in SDGVM has no carbon fluxes, with hydrology simulated by assuming no vegetation cover and a soil depth of 5 cm.
ORCHIDEE	The urban land-cover type is distributed between bare soil and grassland, with bare soil having no carbon fluxes.
IBIS	Urban land is treated as bare soil with no carbon fluxes or vegetation growth.

Supplementary Table 2 Information on global ISA products used in the study.

Dataset	Spatial resolution	Temporal resolution	Available period
GISA2.0	30 m	annual	1985–2019 (35 years)
GAUD	30 m	annual	1985–2016 (32 years)
GAIA	30 m	annual	1985–2018 (34 years)
ESA CCI	300 m	annual	1992–2020 (28 years)

Note: GISA: global impervious surface area product from ref. [27]; GAUD: global annual urban dynamics product from ref. [10]; GAIA: global artificial impervious area from ref. [28]; ESA CCI: the ESA-CCI land cover product[29].

Supplementary Table 3 Information on field observations from ISA studies collected in this study.

Reference No.	Cities	Countries	Sampling Depth (cm)	SOC under ISA (kg C m^{-2})	SOC under green areas (kg C m^{-2})
[30]	Nanjing	China	0–20	2.35	4.52
[31]	New York	USA	0–15	2.29	5.67
[31]	New York	USA	45–60	0.61	3.97
[32]	Lahti	Finland	0–100	1.20	31.8
[33]	Guangzhou	China	0–20	2.69	3.25
[33]	Shenzhen	China	0–20	1.43	4.53
[34]	New York	USA	0–30	2.90	11.3
[34]	New York	USA	30–100	6.70	14.5
[34]	Paris	France	0–30	3.40	9.9
[35]	Seoul	Korea	0–100	2.69	7.84
[35]	Seoul	Korea	0–100	3.13	7.96
[35]	Seoul	Korea	0–100	1.37	5.43
[36]	Auburn	USA	10–30	3.24	6.91
[36]	Auburn	USA	10–30	2.89	6.91
[37]	Urumqi	China	0–80	5.36	8.08
[38]	Yixing	China	0–20	2.46	7.59
[39]	Alabama & Georgia	USA	0–10	1.25	3.38
[39]	Alabama & Georgia	USA	10–20	0.78	1.88
[40]	Urumqi	China	0–100	5.74	8.69
[41]	Nanjing	China	0–20	1.77	4.17
[42]	Toruń	Poland	20–100	3.23	8.85
[43]	Shanghai	China	0–100	4.77	9.91

Supplementary Table 4 Information on permanent bare fallow sites (data compiled from ref. [44]).

Site	Askov B3	Askov B4	Grignon	Kursk	Rothamsted	Ultuna	Versailles
Country	Denmark	Denmark	France	Russia	UK	Sweden	France
Longitude/Latitude	55.28N<br>9.07E	55.28N<br>9.07E	48.51N<br>1.55E	51.73N<br>36.19E	51.82N<br>0.35E	59.49N<br>17.38E	48.48N<br>2.08E
Bare-fallow starting year	1956	1956	1959	1965	1959	1956	1928
Last sampling year	1985	1985	2007	2001	2008	2007	2008
Monitoring period (yr)	29	30	50	37	49	52	81
Former land cover type	Arable	Arable	Grassland	Arable	Grassland	Arable	Grassland
Initial bulk density (g cm^{-1})	1.50	1.50	1.20	1.13	0.94	1.44	1.30
Final bulk density (g cm^{-1})	1.50	1.50	1.21	1.13	1.43	1.43	1.44
Sampling depth (cm)	20	20	25	25	23	20	25

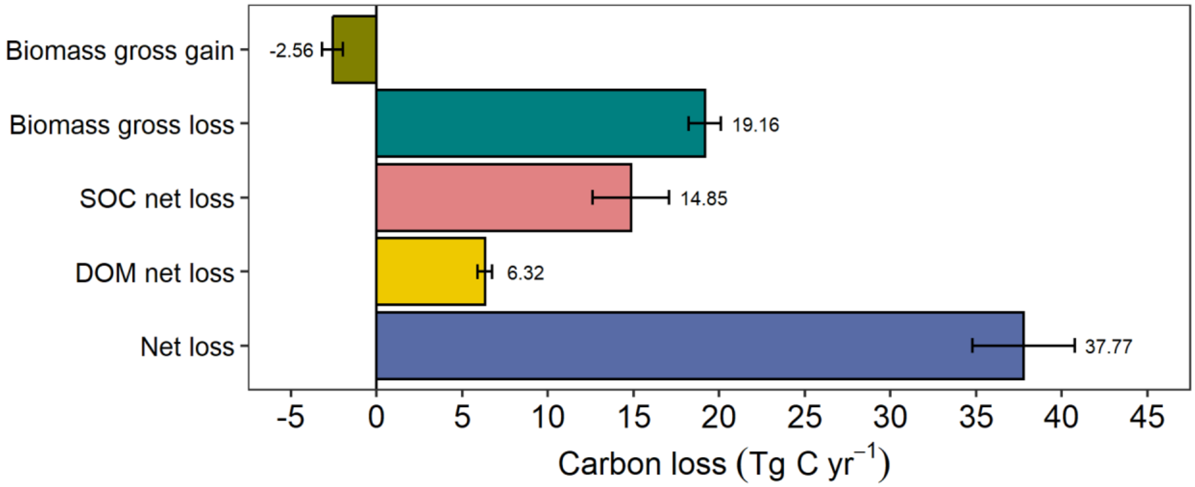

Supplementary Fig. 1 Carbon stock changes due to settlement expansion reported by Annex I countries in NGHGIs for 1993–2018. SOC means organic carbon stocks in mineral soil; DOM means organic carbon stocks in dead organic matter including surface litter and dead wood. For carbon stock in SOC and DOM, only net changes were reported by NGHGIs. The net loss is the sum of biomass gross gain, biomass gross loss, SOC net loss, and DOM net loss. The error bars indicate one standard error.

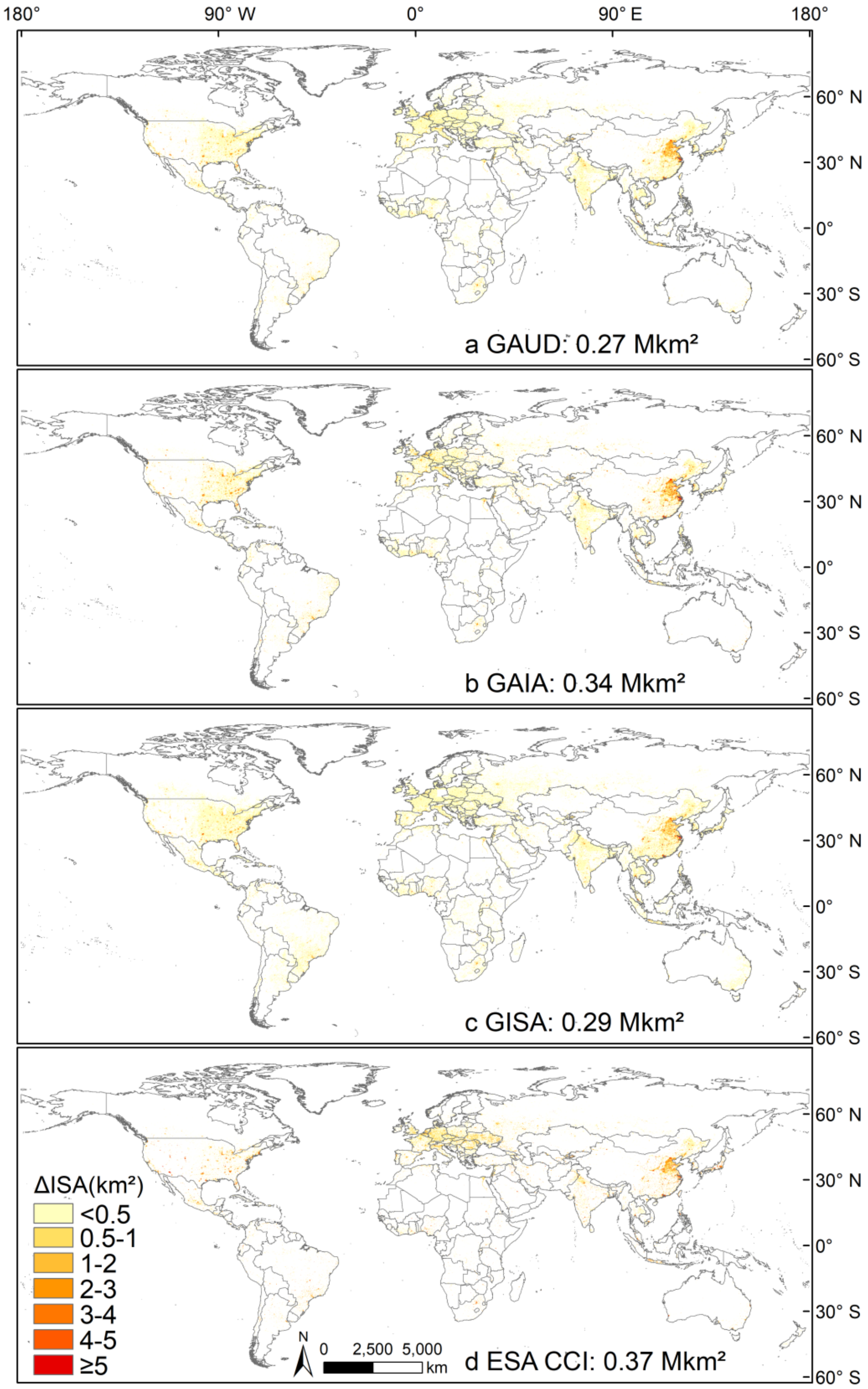

Supplementary Fig. 2 The spatial distributions of the cumulative ISA expansion during the overlapping period 1996–2015 for the four ISA products at a resolution of 5 km. a, GAUD (0.27 Mkm^2); **b**, GAIA (0.34 Mkm^2); c, GISA (0.29 Mkm^2); and **d,** ESA CCI (0.37 Mkm^2).

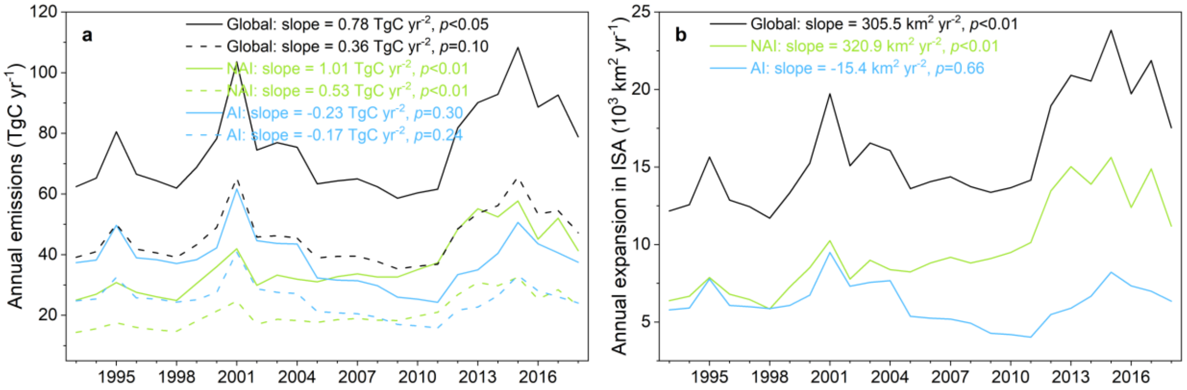

Supplementary Fig. 3 Annual carbon emissions and the corresponding ISA expansions (ΔISA) for the globe, Annex I (AI) and non-Annex I (NAI) countries, respectively. a, Annual carbon emissions with the upper (solid lines) and lower (dashed lines) boundaries for the globe (black), AI (blue), and NAI (green) countries over 1993–2018; **b**, Corresponding ISA expansions for the globe, AI, and NAI countries over 1993–2018. The slopes derived from the linear regressions with time and *p*-values were shown in the panels.

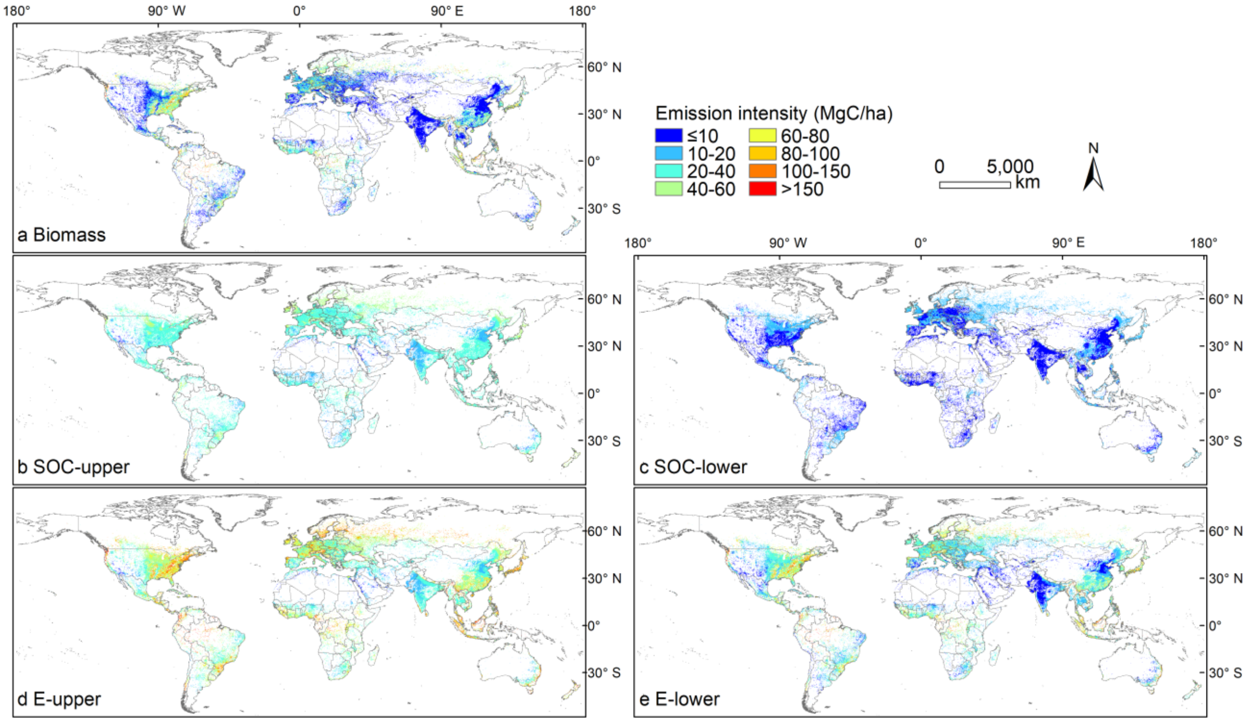

Supplementary Fig. 4 Carbon emissions per area of ISA expansion over 1993–2018 at a resolution of 5 km. a, Biomass emissions (including live biomass, surface litter and dead wood); **b,** SOC emissions with upper boundary; **c,** SOC emissions with lower boundary; **d,** Total emissions with upper boundary; and **e,** Total emissions with lower boundary.

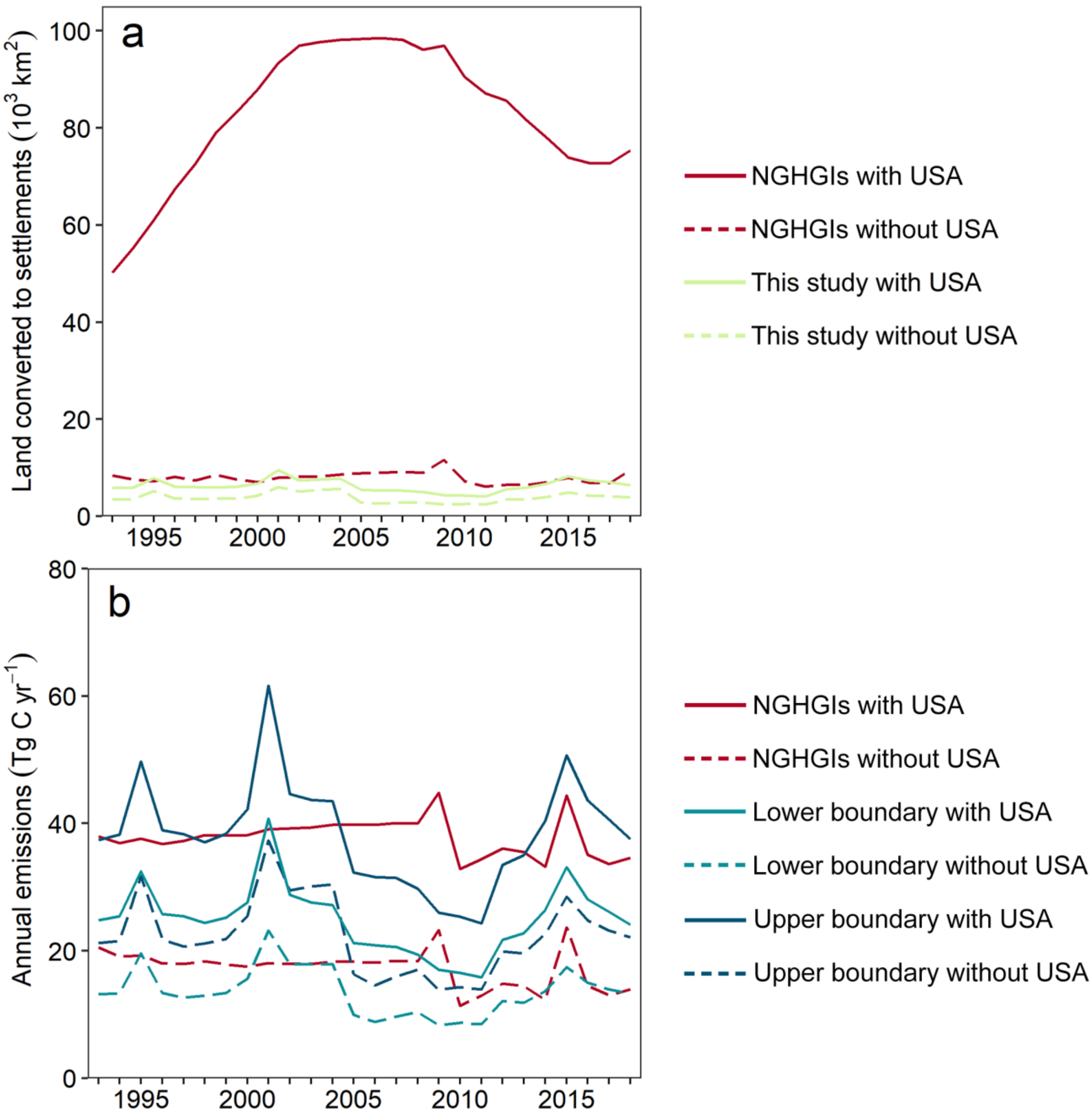

Supplementary Fig. 5 Land conversions to ISA or settlement and the associated carbon losses for Annex I countries. a, Land area converted to ISA in this study and land area converted to settlement reported by NGHGIs for Annex I countries, with data being shown with and without the USA, respectively; **b,** Annual carbon losses (biomass and SOC) from ISA or settlement expansion estimated in this study for lower and upper boundaries and reported by NGHGIs, with and without the USA, respectively.

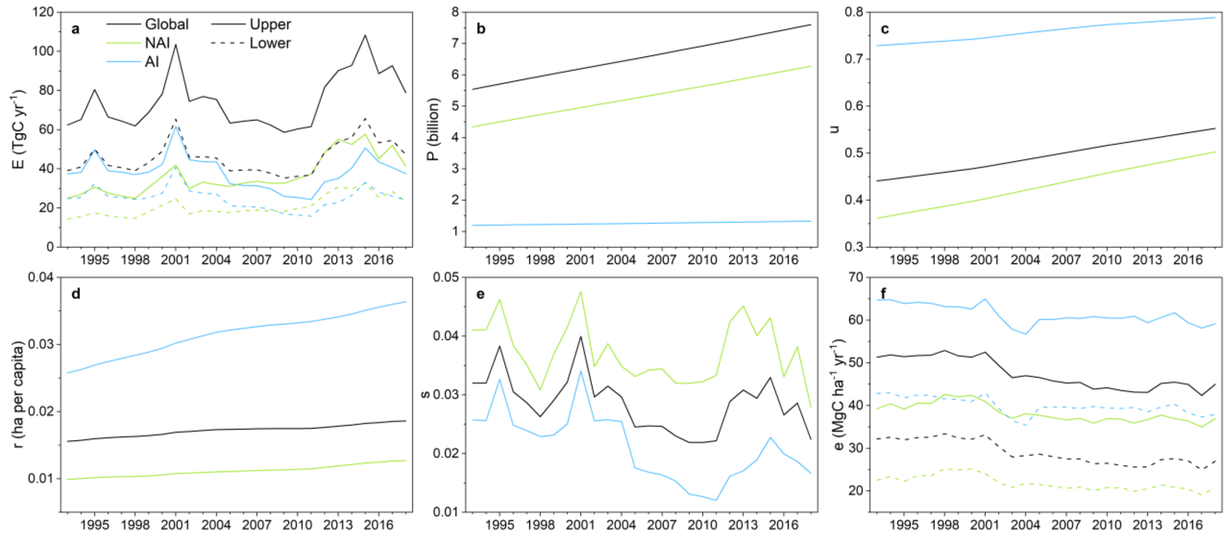

Supplementary Fig. 6 Annual dynamics of E and the underlying drivers during 1993–2018 for the globe, Annex I (AI) and non-Annex I (NAI) countries, respectively. **a**, ISA-driven carbon emissions; **b**, total population P; **c**, urbanization rate u; **d**, residential ISA intensity r; **e**, ISA expansion speed-up factor s; and **f**, the carbon emission intensity e. The black, blue and green lines indicate time-series for the globe, AI and NAI countries, respectively, whereas the solid and dashed lines represent time-series for the upper and lower boundary in panels **a** and **f**.

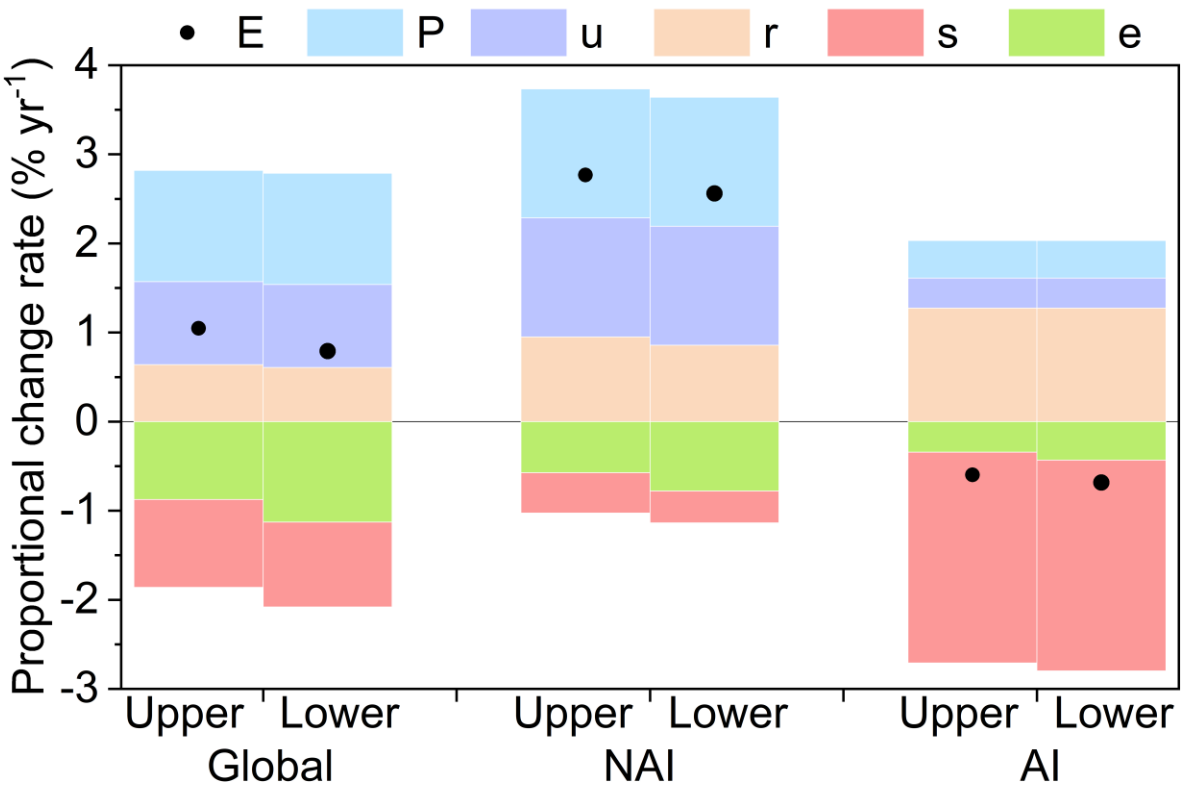

Supplementary Fig. 7 Drivers of carbon emissions due to ISA expansion over 1993–2018 using the "ISA-driven Emissions Identity" framework. Relative change rates in ISA-driven carbon emissions (*E*) with the upper and lower boundary and the underlying drivers (total population *P*, urbanization rate *u*, residential ISA intensity *r*, ISA expansion speed-up factor *s*, and the carbon emission intensity *e*) are shown for the global total, the non-Annex I countries (NAI), and the Annex I countries (AI) of the UNFCCC.

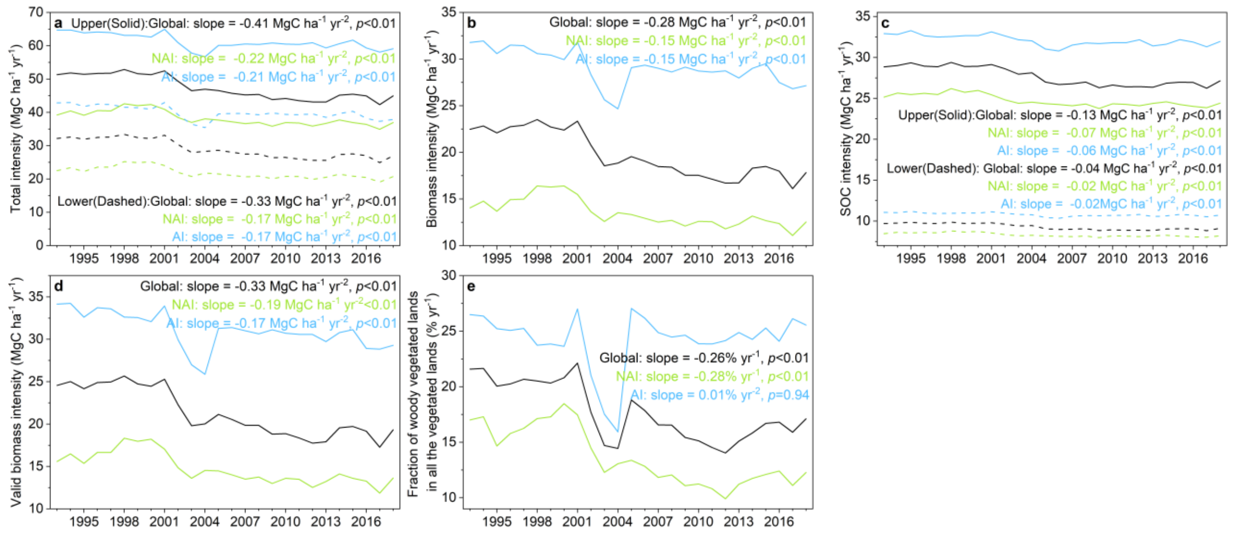

Supplementary Fig. 8 Annual time series of ISA-driven carbon emission intensity over 1993–2018 for the globe, Annex I (AI), and non-Annex I (NAI) countries, respectively. Emission intensity in panels **a**-**c** refers to the carbon emissions per area of ISA expansion by combing all land cover sources. Valid biomass intensity in panel **d** means biomass carbon emission per area of ISA expansion from forest, shrubland, wetland, cropland and grassland for which biomass carbon losses were accounted for. Fraction of biomass sources in panel **e** means the ratio of ISA expansion sourced from woody vegetation (i.e., forest and shrubland) to the total ISA expansion from all vegetated lands (i.e., forest, shrubland, wetland, cropland, and grassland). The black, blue and green lines indicate time-series for the globe, AI and NAI countries, respectively, whereas the solid and dashed lines represent time series for the upper and lower boundary in panels **a** and **c**. The slopes and the *p*-values derived from the linear regressions are shown.

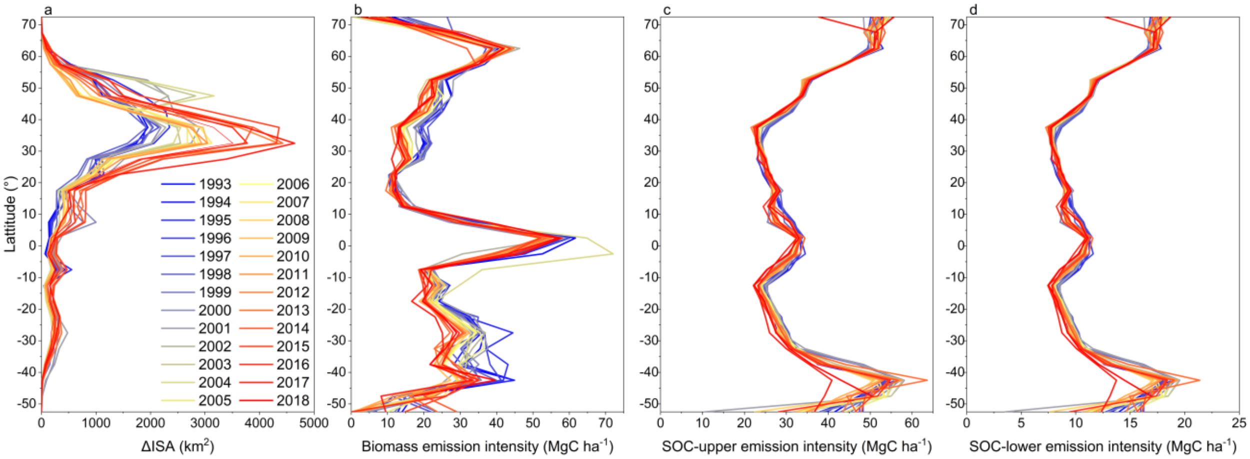

Supplementary Fig. 9 The latitudinal distribution of annual ISA expansion and the associated carbon emissions with a gradient of 5º resolution for each year of 1993–2018. a, ISA expansion (ΔISA). **b**, biomass emission intensity. **c-d**, SOC emission intensity with the upper and lower boundary, respectively.

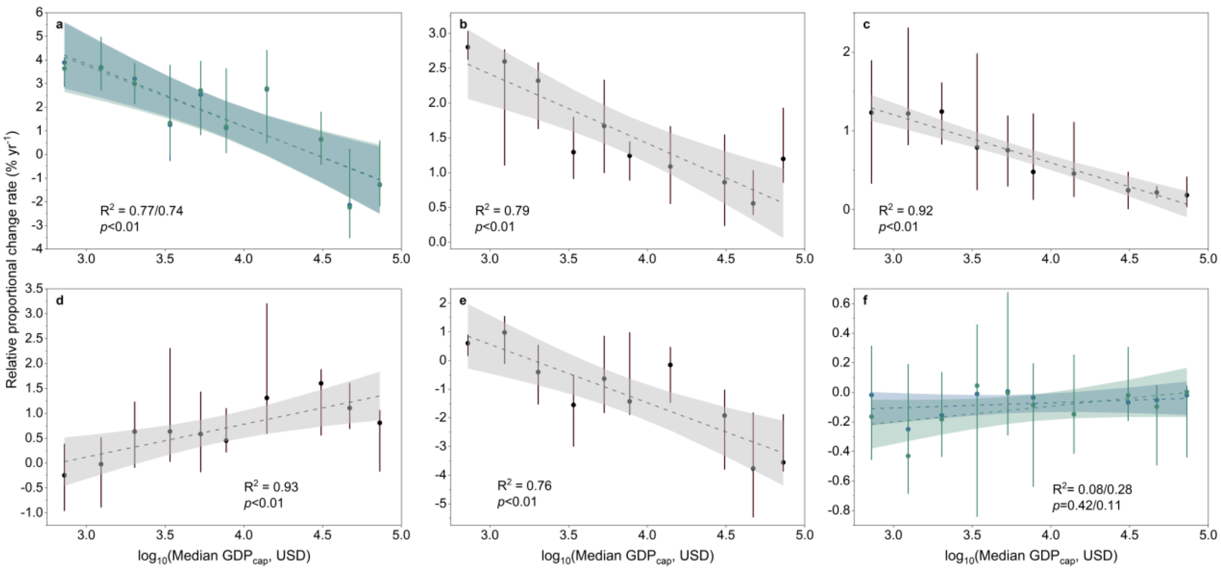

Supplementary Fig. 10 Relationships between relative proportional change rates in ISA-driven carbon emissions, and the underlying drivers over 1993–2018, and per capita GDP in 2018. a, The relative change rate in carbon emissions (*E*). **b–f**, The relative change rates in total population *P*, urbanization rate *u*, residential ISA intensity *r*, ISA expansion speed-up factor *s*, and the carbon emission intensity *e*, respectively. Solid dots represent the median values of proportional change rates in the carbon emissions and their driving factors over 1993–2018 for the ten different country groups of increasing per capita GDP (GDP_{cap}, see Methods), with vertical lines showing 25%–75% percentiles. The horizontal axis shows the logarithmically transformed median values of GDP_{cap} in 2018 for each country group. The dashed lines represent fitted linear regressions with the shading indicating the 95% confidence intervals. For panels **a** and **f**, light blue indicates the results using the upper-boundary emissions, while light green indicates those with the lower-boundary emissions.

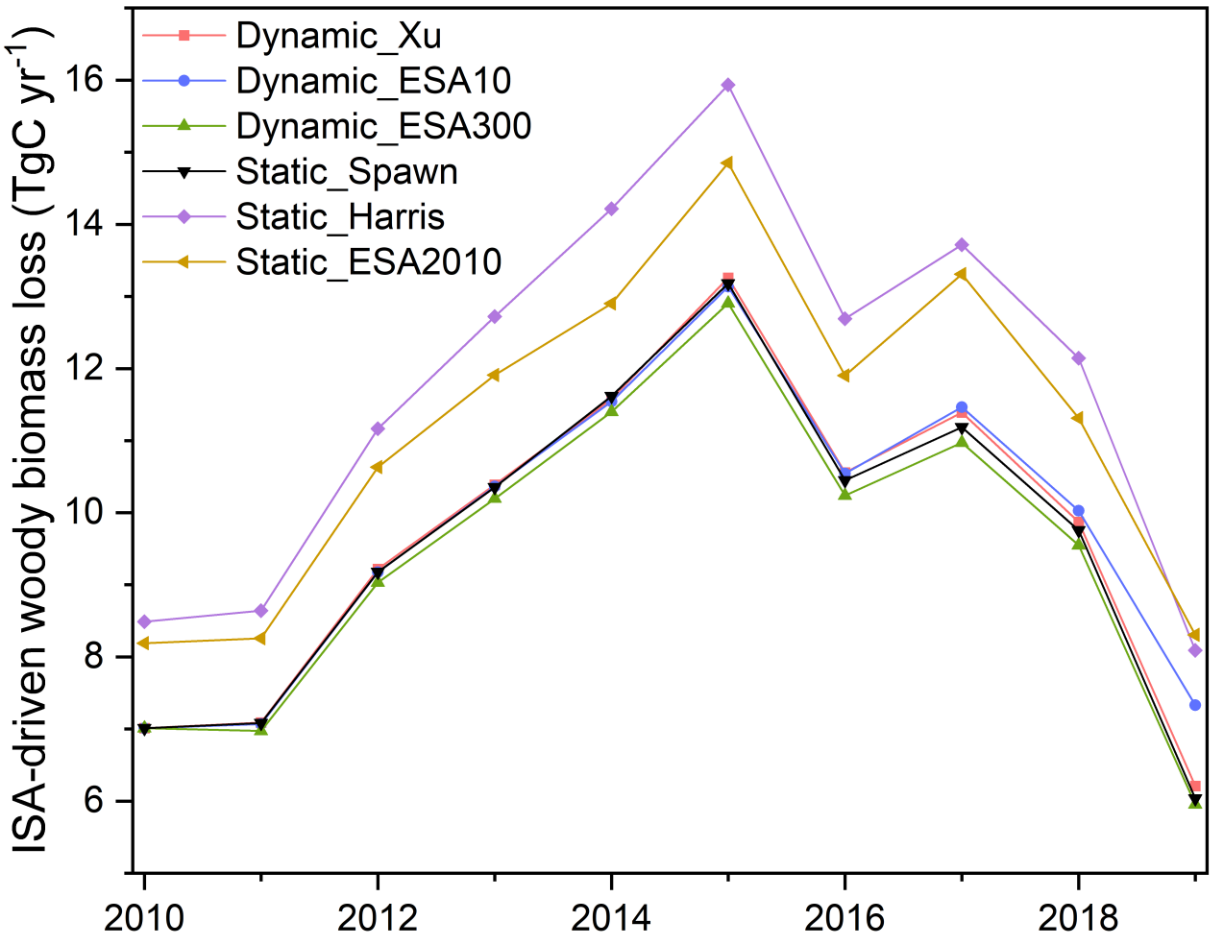

Supplementary Fig. 11 ISA-driven woody biomass carbon emissions using the average ISA expansion of the four ISA products (i.e., GAUD, GAIA, GISA and ESA CCI) based on different dynamic and static biomass density maps during 2010–2019. 'Dynamic_Xu' indicates the emissions derived using the dynamic biomass density maps generated through integrating the static biomass density map from ref. [20] and the dynamics maps from ref. [45]; 'Dynamic_ESA10' indicates the emissions derived using the dynamic biomass density maps generated through integrating the static biomass density map from ref. [20] and dynamics maps from ESA CCI[22] but with first resampling of 10-km, whereas 'Dynamic_ESA300' is similar to Dynamic_ESA10 but with first resampling of 300-m. 'Static_Spawn', 'Static_Harris' and 'Static_ESA2010' indicate the emissions derived using the static biomass density maps from ref. [20], ref. [21], and ref. [22] in 2010, respectively.

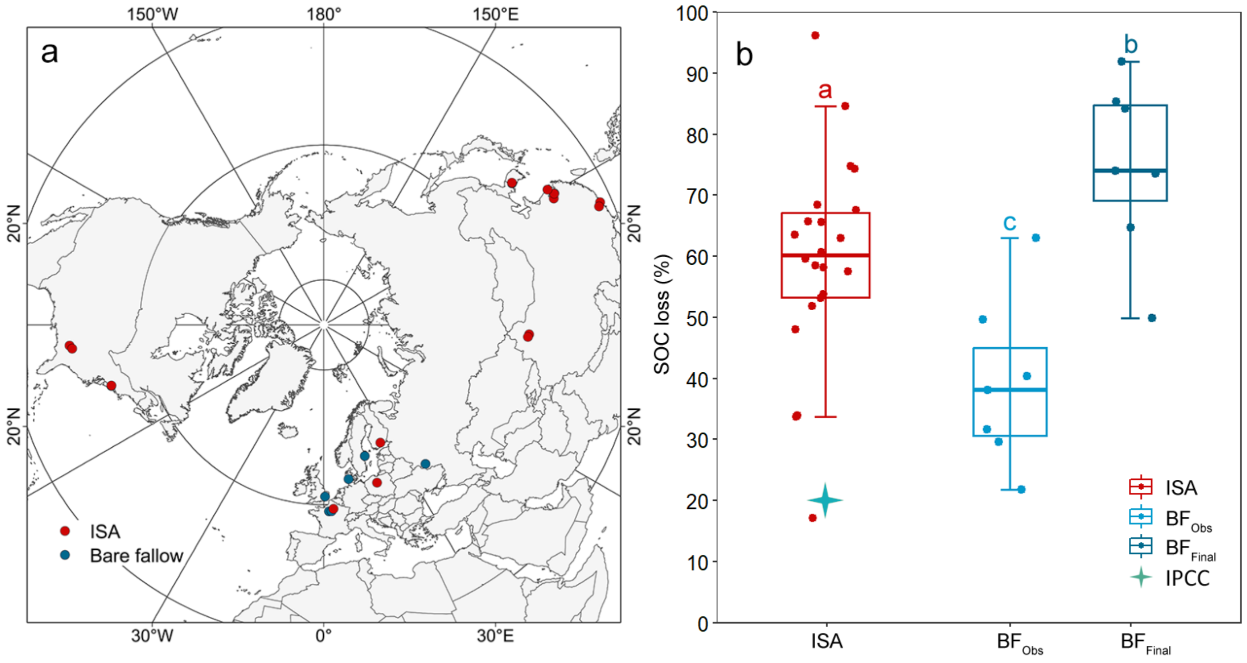

Supplementary Fig. 12 Soil organic carbon loss (%) following ISA establishment or under long-term bare fallow. a, The spatial distribution of observation sites for ISA and bare fallow studies; **b**, Percentages of SOC loss under ISA and bare fallow. In subplot **b**, SOC loss ratio under ISA (59.5%) was obtained by meta-analysis compiled in this study, while the Tier 1 approach in IPCC guidelines adopts 20% SOC loss ratio as default. BF_{obs} and BF_{final} indicate SOC loss under bare fallow at the time of last observation and predicted SOC loss when SOC finally stabilizes, respectively. Different letters indicate statistically significant differences among groups as determined by t-test (*p*<0.05; for ISA, n=22; for bare fallow, n=7). The boxes show the inner 50% percentiles, with the vertical center lines indicating the median values. The lower and upper whiskers denote the lowest and highest values within 1.5 times interquartile ranges, respectively.

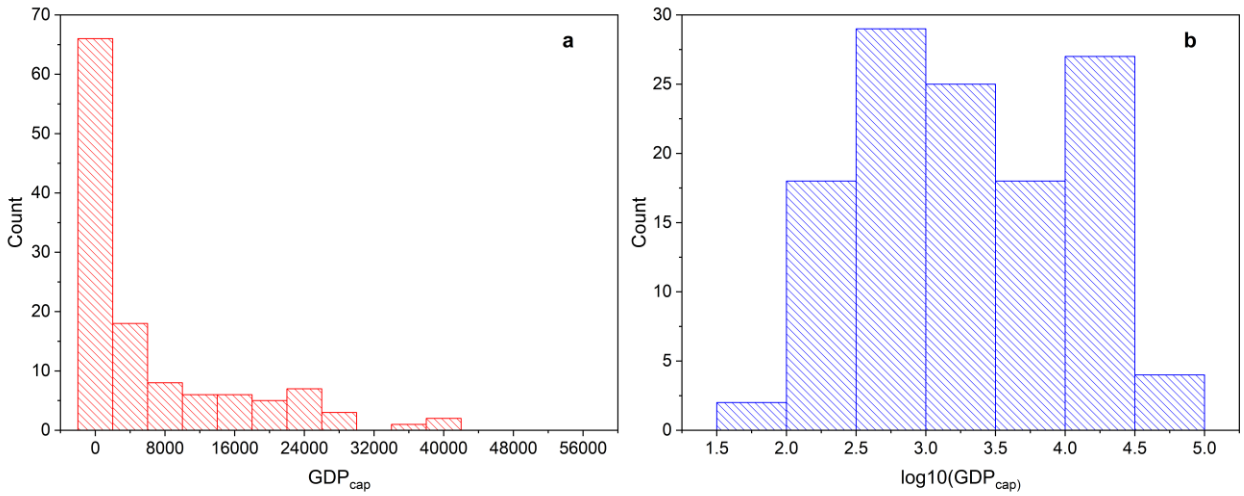

Supplementary Fig. 13 Histograms of per capita GDP (GDP_{cap}) in 1993 at the country scale. a, the original GDP_{cap}, and **b**, the logarithm transformation using 10 as the base.

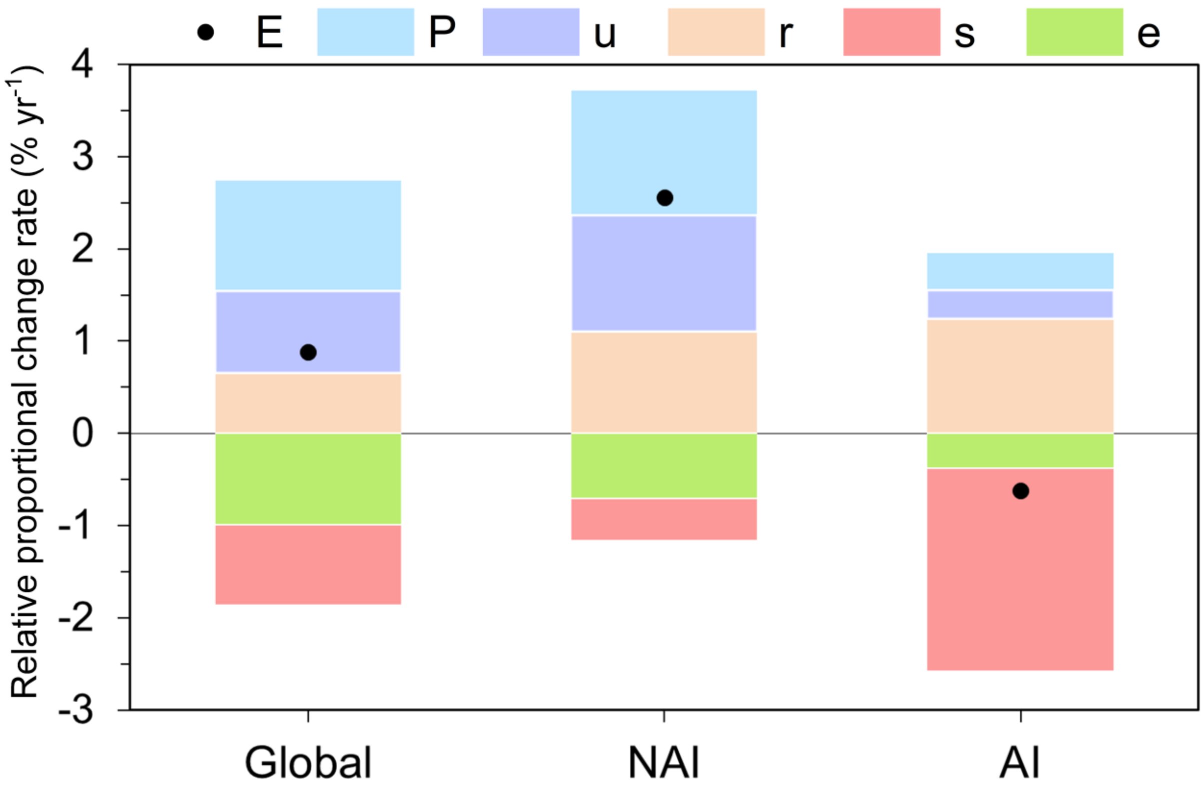

Supplementary Fig. 14 Drivers of carbon emissions due to ISA expansion over 1993–2018 using the LMDI decomposition approach. The average values of the decomposition results for ISA-driven carbon emissions of the upper- and lower-boundary estimations are shown for the global total, the non-Annex I countries (NAI), and the Annex I countries (AI) of the UNFCCC. The underlying drivers are total population *P*, urbanization rate *u*, residential ISA intensity *r*, ISA expansion speed-up factor *s*, and the carbon emission intensity *e*.

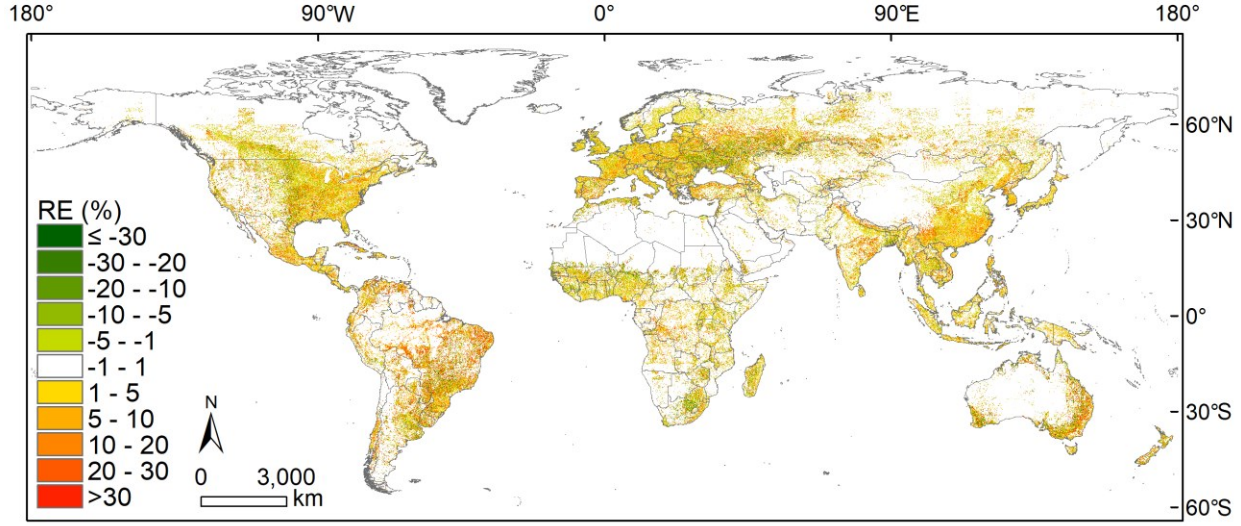

Supplementary Fig. 15 Spatial distribution of RE (relative error, in %) for biomass density interpolation at a 5-km grid. The RE values indicate the uncertainty referring to biomass density interpolation.

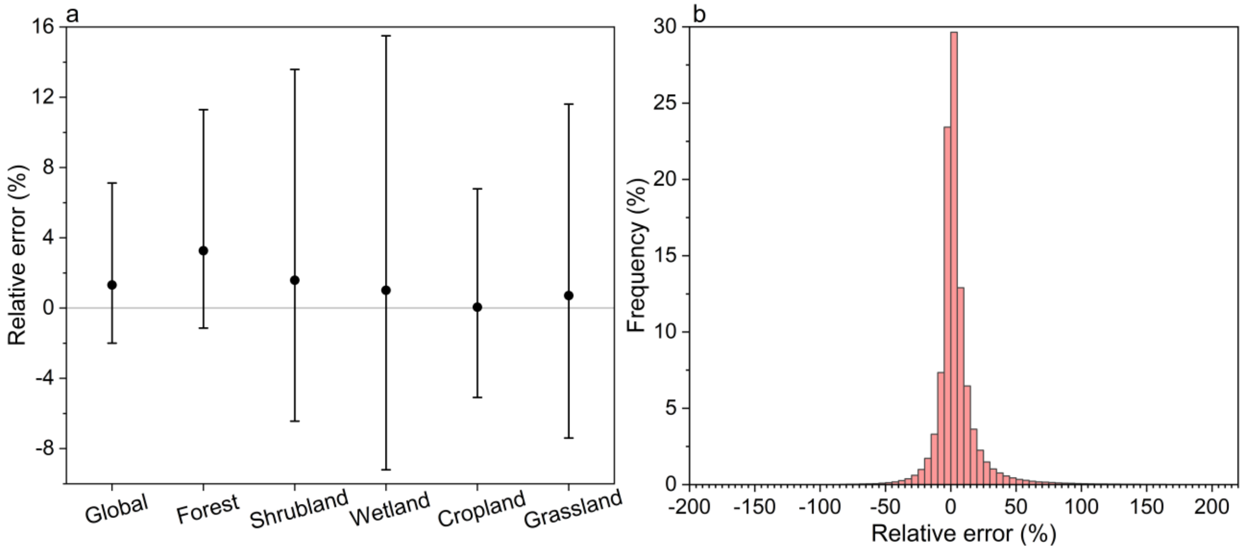

Supplementary Fig. 16 The RE (relative error, %) of biomass density interpolation for the buffer ISA-free pixels at a global scale and for different land cover types (a) and its histogram (b). The dots and the error bars in panel **a** indicate the median value and the interquartile range of RE, respectively.

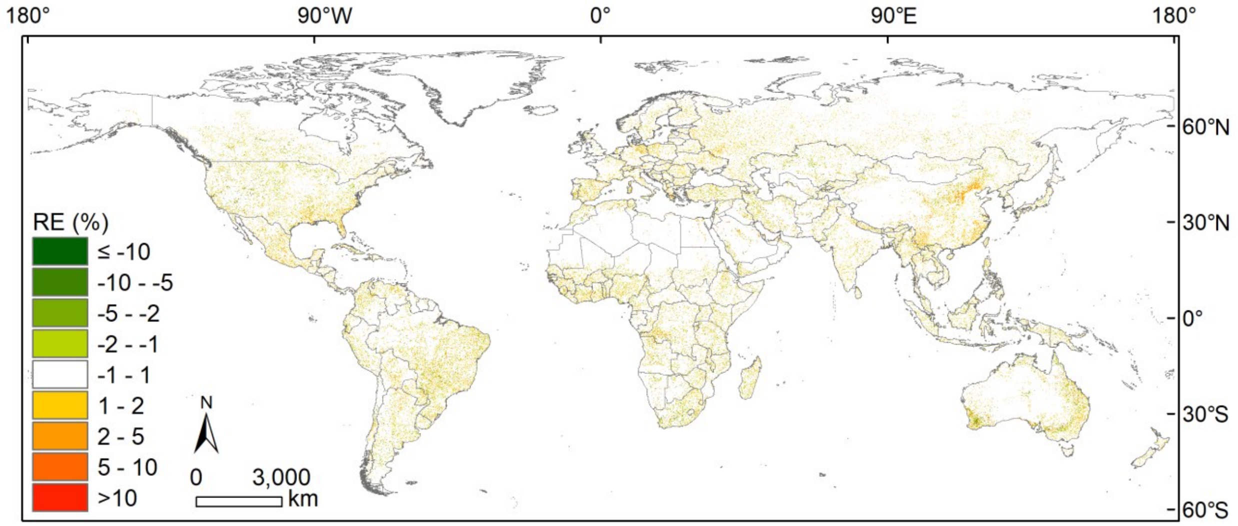

Supplementary Fig. 17 Spatial distribution of RE (relative error, in %) for soil organic carbon (SOC) density interpolation at a 5-km grid. The RE values indicate the uncertainty referring to SOC density interpolation.

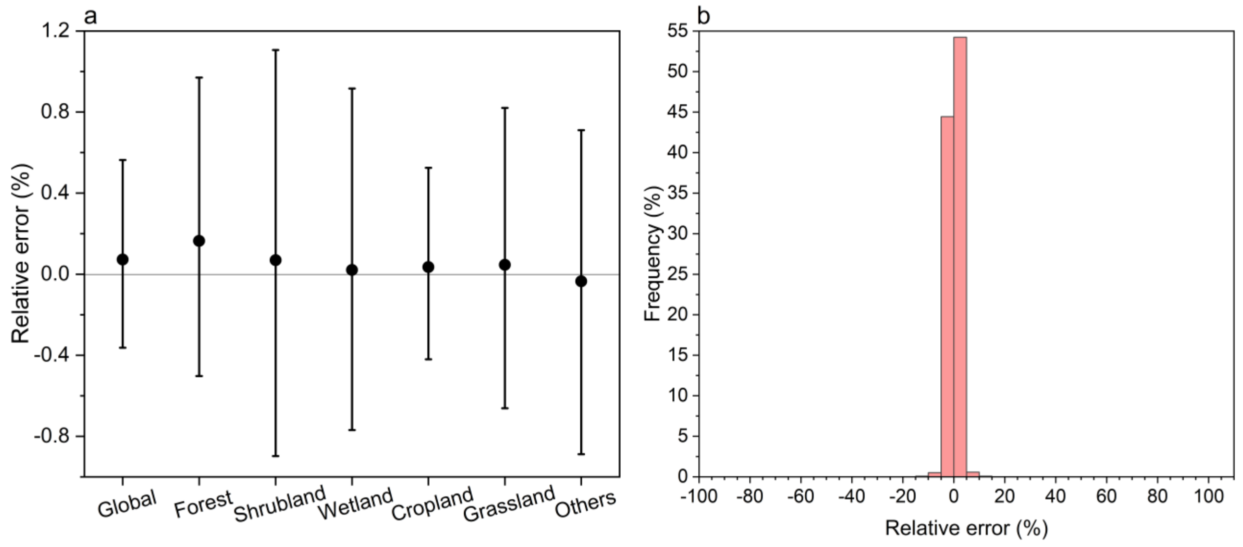

Supplementary Fig. 18 The RE (relative error, %) of SOC interpolation for the buffer ISA-free pixels at a global scale and for different land cover types (a) and its histogram (b). The dots and the error bars in panel **a** indicate the median value and the interquartile range of RE, respectively.

Supplemental References

1. Hurtt, G. C. et al. Harmonization of global land use change and management for the period 850–2100 (LUH2) for CMIP6. Geoscientific Model Development 13, 5425–5464 (2020).
2. Zhang, X. et al. GLC_FCS30: Global land-cover product with fine classification system at 30 m using time-series Landsat imagery. Earth System Science Data 13, 2753–2776 (2021).
3. Winkler, K., Fuchs, R., Rounsevell, M. & Herold, M. Global land use changes are four times greater than previously estimated. Nat Commun 12, 2501 (2021).
4. Zhuang, Q. et al. Impact of global urban expansion on the terrestrial vegetation carbon sequestration capacity. Science of The Total Environment 879, 163074 (2023).
5. Seto, K. C., Güneralp, B. & Hutyra, L. R. Global forecasts of urban expansion to 2030 and direct impacts on biodiversity and carbon pools. Proc. Natl. Acad. Sci. U.S.A. 109, 16083–16088 (2012).
6. Gong, P., Li, X. & Zhang, W. 40-Year (1978–2017) human settlement changes in China reflected by impervious surfaces from satellite remote sensing. Science Bulletin 64, 756–763 (2019).
7. Pesaresi, M. et al. A global human settlement layer from optical HR/VHR RS data: Concept and first results. IEEE Journal of Selected Topics in Applied Earth Observations and Remote Sensing 6, 2102–2131 (2013).
8. KEMPER, T. et al. GHS built-up grid, derived from Landsat, multitemporal (1975, 1990, 2000, 2014), IR2017 V1. 0.
9. Esch, T. et al. Urban Footprint Processor—Fully Automated Processing Chain Generating Settlement Masks From Global Data of the TanDEM-X Mission. IEEE Geosci. Remote Sensing Lett. 10, 1617–1621 (2013).

10. Liu, X. et al. High-spatiotemporal-resolution mapping of global urban change from 1985 to 2015. Nature Sustainability 3, 564–570 (2020).
11. Huang, X. et al. 30 m global impervious surface area dynamics and urban expansion pattern observed by Landsat satellites: From 1972 to 2019. Sci. China Earth Sci. 64, 1922–1933 (2021).
12. Ren, H. et al. Mapping High-Resolution Global Impervious Surface Area: Status and Trends. IEEE Journal of Selected Topics in Applied Earth Observations and Remote Sensing 15, 7288–7307 (2022).
13. Wang, N., Zhang, X., Yao, S., Wu, J. & Xia, H. How Good Are Global Layers for Mapping Rural Settlements? Evidence from China. Land 11, 1308 (2022).
14. Schmidt, S. & Barron, C. Mapping impervious surfaces precisely—a GIS-based methodology combining vector data and high-resolution airborne imagery. Journal of Geovisualization and Spatial Analysis 4, 14 (2020).
15. Feng, X. et al. Integrating Zhuhai-1 Hyperspectral Imagery With Sentinel-2 Multispectral Imagery to Improve High-Resolution Impervious Surface Area Mapping. IEEE Journal of Selected Topics in Applied Earth Observations and Remote Sensing 15, 2410–2424 (2022).
16. Flanagan, M. & Civco, D. L. Subpixel impervious surface mapping. in Proceedings of the 2001 ASPRS Annual Convention vol. 23 (Bethesda, MD: American Society for Photogrammetry & Remote Sensing St. Louis, MO, 2001).
17. Zhang, X. et al. Development of a global 30 m impervious surface map using multisource and multitemporal remote sensing datasets with the Google Earth Engine platform. Earth System Science Data 12, 1625–1648 (2020).

18. Meijer, J. R., Huijbregts, M. A., Schotten, K. C. & Schipper, A. M. Global patterns of current and future road infrastructure. Environmental Research Letters 13, 064006 (2018).

19. Ramm, F., Topf, J. & Chilton, S. OpenStreetMap: Using and Enhancing the Free Map of the World. (UIT Cambridge Cambridge, 2011).

20. Spawn, S. A., Sullivan, C. C., Lark, T. J. & Gibbs, H. K. Harmonized global maps of above and belowground biomass carbon density in the year 2010. Sci Data 7, 112 (2020).

21. Harris, N. L. et al. Global maps of twenty-first century forest carbon fluxes. Nat. Clim. Chang. 11, 234–240 (2021).

22. Santoro, M. & Cartus, O. ESA Biomass Climate Change Initiative (Biomass_cci): Global datasets of forest above-ground biomass for the years 2010, 2017, 2018, 2019 and 2020, v4. 5183 Files, 302039459020 B [object Object] https://doi.org/10.5285/AF60720C1E404A9E9D2C145D2B2EAD4E (2023).

23. Ang, B. W. The LMDI approach to decomposition analysis: a practical guide. Energy Policy 33, 867–871 (2005).

24. Moutinho, V., Madaleno, M., Inglesi-Lotz, R. & Dogan, E. Factors affecting CO2 emissions in top countries on renewable energies: a LMDI decomposition application. Renewable and Sustainable Energy Reviews 90, 605–622 (2018).

25. Ma, X. et al. Carbon emissions from energy consumption in China: Its measurement and driving factors. Science of the total environment 648, 1411–1420 (2019).

26. Xia, Q., Wang, H., Liu, X. & Pan, X. Drivers of global and national CO2 emissions changes 2000–2017. Climate Policy 21, 604–615 (2021).

27. Huang, X. et al. Toward accurate mapping of 30-m time-series global impervious surface area (GISA). International Journal of Applied Earth Observation and Geoinformation 109, 102787 (2022).
28. Gong, P. et al. Annual maps of global artificial impervious area (GAIA) between 1985 and 2018. Remote Sensing of Environment 236, 111510 (2020).
29. Bontemps, S. et al. Consistent global land cover maps for climate modelling communities: current achievements of the ESA's land cover CCI. in Proceedings of the ESA living planet symposium, Edimburgh vol. 13 9–13 (2013).
30. Wei, Z., Wu, S., Zhou, S., Li, J. & Zhao, Q. Soil Organic Carbon Transformation and Related Properties in Urban Soil Under Impervious Surfaces. Pedosphere 24, 56–64 (2014).
31. Raciti, S. M., Hutyra, L. R. & Finzi, A. C. Depleted soil carbon and nitrogen pools beneath impervious surfaces. Environmental Pollution 164, 248–251 (2012).
32. Lu, C., Kotze, D. J. & Setälä, H. M. Soil sealing causes substantial losses in C and N storage in urban soils under cool climate. Science of the Total Environment 725, 138369 (2020).
33. Dou, X., Lu, M. & Chen, L. Comparison of soil organic carbon and nitrogen dynamics between urban impervious surfaces and vegetation. Land Degrad Dev 32, 5455–5467 (2021).
34. Cambou, A. et al. Estimation of soil organic carbon stocks of two cities, New York City and Paris. Science of The Total Environment 644, 452–464 (2018).
35. Bae, J. & Ryu, Y. High soil organic carbon stocks under impervious surfaces contributed by urban deep cultural layers. Landscape and Urban Planning 204, 103953 (2020).
36. Majidzadeh, H., Lockaby, B. G., Price, R. & Governo, R. Soil Carbon and Nitrogen Dynamics beneath Impervious Surfaces. Soil Science Society of America Journal 82, 663–670 (2018).

37. Yan, Y., Kuang, W., Zhang, C. & Chen, C. Impacts of impervious surface expansion on soil organic carbon – a spatially explicit study. Sci Rep 5, 17905 (2015).
38. Wei, Z., Wu, S., Yan, X. & Zhou, S. Density and Stability of Soil Organic Carbon beneath Impervious Surfaces in Urban Areas. PLOS ONE 9, 7 (2014).
39. Majidzadeh, H., Lockaby, B. G. & Governo, R. Effect of home construction on soil carbon storage-A chronosequence case study. Environmental Pollution 226, 317–323 (2017).
40. Yan, Y., Zhang, C., Kuang, W., Luo, G. & Chen, C. Orgnic carbon charasteristics of soils beneath urban impervious surface in northern Tianshan urban cluster (in Chinese). Progress in Geography 34, 781–789 (2015).
41. Chen, H. et al. Simulating effects of urban soil sealing on the soil organic carbon pool (in Chinese). Acta Ecologica Sinica 37, 2600–2610 (2017).
42. Piotrowska-Długosz, A. & Charzyński, P. The impact of the soil sealing degree on microbial biomass, enzymatic activity, and physicochemical properties in the Ekranic Technosols of Toruń (Poland). J Soils Sediments 15, 47–59 (2015).
43. Zhang, Q., Liang, J., Wu, H., Zheng, S. & Huang, J. Effect of land use change on soil organic carbon pool in process of urbanization: a case study of Sanlin Green Wedge, Shanghai (in Chinese). Acta Agriculturae Zhejiangensis 33, 1062–1068 (2021).
44. Barré, P. et al. Quantifying and isolating stable soil organic carbon using long-term bare fallow experiments. Biogeosciences 7, 3839–3850 (2010).
45. Xu, L. et al. Changes in global terrestrial live biomass over the 21st century. Science Advances 7, eabe9829 (2021).
